# How climate change shapes global systemic risk transmission: A complex network approach

**DOI:** 10.1371/journal.pone.0337401

**Published:** 2026-05-20

**Authors:** Li Zeng, Wee-Yeap Lau

**Affiliations:** 1 School of Finance and Economics, Henan Polytechnic University, Jiaozuo, Henan, China; 2 Faculty of Business and Economics, Universiti Malaya, Kuala Lumpur, Malaysia; Bucharest University of Economic Studies: Academia de Studii Economice din Bucuresti, ROMANIA

## Abstract

This study investigates the dynamic impact of climate change performance on extreme tail risk transmission across global financial markets. Based on the “Too Extreme to Fail” conceptual framework, we propose a cascading failure network model using QRNN-∆CoVaR and QRNN-∆CoES to quantify the domino effect of tail risk propagation. The model captures tail dependencies and reveals how variations in climate governance performance modulate the intensity and pathways of risk contagion. Our main analysis utilizes daily market data from 1998 to 2024, aligned with the Climate Change Performance Index data from 2007 through a matched time window approach. The findings demonstrate that climate-sensitive factors significantly amplify systemic vulnerabilities, whereas superior climate governance serves as a critical risk buffer during periods of extreme volatility. Empirical results reveal significant spatial and temporal heterogeneity in risk contribution, with certain regions exhibiting higher sensitivity and momentum during major financial crises. Backtesting results confirm that our proposed nonlinear framework provides superior accuracy in quantifying global systemic risks compared to traditional linear methods, offering a robust tool for climate-integrated financial stability monitoring.

## 1 Introduction

With the increasing frequency and intensity of global climate change [1], major issues such as loss of life, health problems, significant crop failures, wetland destruction, and energy crises are becoming more frequent. Global climate change has become one of the major challenges facing humanity [2,3]. According to data released by the National Climate Center, more than 200 major climate-related disasters occurred globally in 2023, highlighting the worsening state of global climate security [4–6]. Research shows that climate change is closely linked to overall economic performance, and financial market volatility is inseparable from climate change [7–9]. On the one hand, physical risks triggered by climate change, such as extreme weather, bring high volatility to financial markets [10,11]. On the other hand, extreme climate events often influence financial markets through investor sentiment [12], affecting how investors perceive climate risks [13], triggering market panic [14], increasing irrational behavior [15], influencing investment strategies [16], reducing market returns, and aggravating systemic financial risks [10,17].

In recent years, the spread of global systemic risks has been further exacerbated by frequent major geopolitical conflicts and security events [18], such as the outbreak of the COVID-19 pandemic [19]. Therefore, it is necessary to assess the systemic risk contribution (SRC) of global stock markets [20], analyze the spillover effects and transmission pathways of extreme risks driven by climate change, identify Systemically Important Markets (SIM), provide early warnings of potential risks, mitigate existing risks, and offer insights for investors and policymakers to address future uncertainties [21,22]. Furthermore, this study aims to investigate the impact of climate change on global systemic risk transmission and propose a new approach to analyzing the role of climate factors in extreme risk contagion.

In financial markets, understanding and quantifying the dynamic characteristics of risk contagion is essential [22], especially against the backdrop of increasing systemic risks [23]. Traditional methods, such as Value-at-Risk (VaR) and Conditional Value-at-Risk (CoVaR), are widely used to capture risks under extreme market conditions [24], but they typically rely on the assumption of normal distribution, which may fail to fully depict the complex contagion mechanisms of nonlinear and tail risks [25]. Moreover, these models are less sensitive to changes in market conditions and have limitations when dealing with complex market risks [26]. To more comprehensively capture dynamic and heterogeneous risk transmission in financial markets, we adopt QRNN-∆CoVaR (Quantile Regression Neural Network-based Conditional Value-at-Risk) and QRNN-∆CoES (Quantile Regression Neural Network-based Conditional Expected Shortfall). QRNN-∆CoVaR captures spillover effects between different markets through nonlinear mechanisms, surpassing the linear framework of traditional VaR models [27].

Meanwhile, QRNN-∆CoES measures tail risks through conditional expected losses during extreme events, providing a more accurate estimate of risk contagion. These models not only handle high-dimensional data but also demonstrate greater robustness in addressing complex market volatility. The models proposed in this study are well-suited to capturing nonlinear characteristics and tail risk, making them ideal for analyzing the dynamics of climate-driven risk transmission.

In recent years, frameworks for measuring systemic risk have primarily focused on two perspectives: “Too Big to Fail” [28] and “Too Connected to Fail” [29]. However, these traditional perspectives primarily emphasize the size and interconnectedness of financial entities, which may not fully capture the critical role of extreme events and tail risks in the process of risk contagion [30]. Extreme events, which are rare but highly impactful fluctuations in financial markets, often amplify and spread through the entire system via nonlinear and complex contagion mechanisms [31]. In this context, relying solely on metrics of size or interconnectedness may underestimate the risk contributions of certain entities under extreme conditions [32]. Therefore, this study introduces a new theoretical perspective, “Too Extreme to Fail” to address the limitations of traditional views and to help identify those key entities that could trigger systemic crises under extreme market conditions [33,34]. In the context of this study, “Too Extreme to Fail” refers to a market’s systemic importance arising from its ability to amplify and transmit extreme tail-risk shocks, rather than from its size or degree of interconnectedness under normal conditions. A market is considered “Too Extreme to Fail” if, during extreme downside states, shocks originating from this market generate disproportionately large conditional tail-risk spillovers to other markets. The term “Too Extreme to Fail” is proposed in this study as a conceptual extension of existing systemic risk paradigms. While prior frameworks emphasize size or network centrality under normal market conditions, the proposed concept highlights systemic importance that emerges specifically under extreme tail risk scenarios. To the best of our knowledge, this term has not been formally defined in the existing literature and is introduced here to capture tail-driven systemic relevance. Consequently, this study further accurately calculates the SRC of major global markets [35]. A new SRC formula is proposed, integrating ∆CoVaR [36] and ∆CoES [37] into the calculation framework to more comprehensively reflect the transmission pathways of systemic risks under different conditions. The new formula synthesizes the spillover effects of both normal market conditions and extreme tail risks, ensuring that the measurement of risks considers both routine market fluctuations and the nonlinear accumulation and transmission characteristics of risks during extreme events.

Based on risk transmission, we construct a global complex risk contagion network [38], where nodes represent different countries or markets, and the weight of the edges reflects the intensity of risk contagion between markets. By using metrics such as degree centrality and betweenness centrality from complex networks, we identify SIM that play a key role in the transmission of climate risks [39]. Under this framework, we further simulate the contagion process of extreme risks: when a market triggers extreme risks due to a climate event, the risk spreads through the network, potentially destabilizing other markets and creating cascading effects [12,32,40]. By analyzing the key nodes and vulnerable nodes in the network structure, we can identify the most fragile markets in climate risk transmission, helping policymakers take timely actions to prevent systemic crises.

Our empirical study is based on market index data from 46 countries and regions, incorporating climate risk factors into the risk contagion network. Daily financial data from 1998 to 2024 capture the dynamics risks of long-term, while climate-related data from 2007 onwards are used to assess the impact of climate factors on systemic risks. This dual dataset strategy allows us to analyze a broader financial risk landscape and changes in risk transmission caused by climate events. The results highlight the key role of Southeast Asian markets as critical nodes in the global risk contagion network. Furthermore, our findings indicate that climate shocks accelerate the spread of tail risks across markets, amplifying systemic vulnerabilities. We adopt a bivariate BEKK-GARCH model to capture co-movements between market prices and use ∆CoVaR and ∆CoES to measure spillover effects. The results of complex network analysis show that the transmission of climate risks across markets is asymmetric, with some markets acting as key intermediaries in the contagion process. Additionally, we find that increased uncertainty in climate policies amplifies market volatility and enhances risk contagion between financial markets.

This study contributes to the literature in three main ways.

(1) Theoretical Contribution: We propose a new framework for measuring SRC, combining ∆CoVaR and ∆CoES, and introduce the concept of “Too Extreme to Fail,” expanding the theoretical perspective of systemic risk analysis. By incorporating nonlinear and tail risk factors, we more comprehensively reveal the complex mechanisms of risk contagion between markets.
(2) Methodological Contribution: We introduce a novel approach that integrates QRNN with traditional risk measurement techniques, combining dynamic network models with tail risk analysis. Based on price time-series data, we quantify the SRC of major markets. Compared with traditional measurement methods, our approach not only provides more accurate results but also captures real-time transmission paths of extreme risks and identifies key nodes within the system.
(3) Empirical Contribution: We conducted a comprehensive analysis of markets across 46 countries and regions, constructing a complex risk network to identify the transmission of systemic risks and reveal how climate risks and extreme events amplify risk contagion in global financial markets. Additionally, our analysis offers policymakers more reliable risk warnings and mitigation strategies, deepens the understanding of systemic risks, and provides practical guidance for future crisis prevention.

The structure of the paper is as follows: The literature review section reviews relevant literature, the data and research methodology section introduces the data and research methodology, the results and discussion section presents results and discussion, and the conclusion section summarizes the main conclusions.

## 2 Literature review

Systemic financial risk is often described as a “perceptible but difficult to define concept” [41]. According to the Guidance to Assess the Systemic Importance of Financial Institutions, Markets, and Instruments jointly issued by the IMF, FSB, and BIS in 2009, systemic financial risk refers to the risk that disruptions within the financial system, whether at a global or local level, could interrupt financial services and have potentially negative impacts on the real economy [42,43]. Current research on systemic financial risk focuses on effective measurement [44], contagion and spillover effects [17], macro-management tools, and regulatory strategies [45].

Accurately measuring systemic financial risk is essential for effective risk management [28]. Existing approaches to systemic risk assessment encompass several key categories [21]. Probabilistic models, also known as signal models, utilize historical data to identify factors that trigger financial crises and estimate the likelihood of future crises [46]. Composite index methods aggregate weighted indicators from multiple sectors into a comprehensive financial stress index, including banking, bonds, foreign exchange, and equities [47–49]. Model methods are widely employed to estimate potential losses for individual assets or markets, such as the traditional VaR [50]. However, newer models like Tail VaR [51] and CoVaR [52] have proven more effective in capturing inter-dependencies and tail risk contagion across financial markets.

As financial systems increase in complexity, linear models have become inadequate in representing the nonlinear contagion mechanisms inherent in tail risks [24]. To address these limitations, recent studies have turned to advanced machine learning [53] and deep learning models [54]. In this study, we employ the QRNN model [55], which combines the nonlinear modeling capabilities of neural networks with the robustness of quantile regression, providing a novel and sophisticated approach to systemic risk analysis. This methodology enhances our ability to capture the intricate dynamics of risk contagion in interconnected financial markets [56].

The highly interconnected nature of financial systems can amplify risk contagion, posing a threat to overall stability [57]. Research on systemic risk contagion has evolved considerably, shifting from early studies that emphasized pairwise interactions among financial entities to more advanced models that capture the overall network structure of financial systems [17,58]. Scholars have since developed methods to map these systems as complex networks [32], allowing for a more accurate depiction of interdependencies. Some studies quantify bilateral relationships between institutions or markets [59], while others examine the interactions between individual institutions and the financial system as a whole [60]. Recent advances in econometric techniques, such as LASSO [61], GLASSO [62], and elastic net regularization [63], have made it possible to construct high-dimensional networks that include numerous markets and institutions, overcoming the “curse of dimensionality”. This study utilizes these advanced methods to construct a complex global network for visualizing risk contagion, enabling a more comprehensive and insightful risk analysis.

Climate change is also becoming a focal point in the study of systemic financial risk transmission [8]. The integration of high-frequency financial data with climate indices enables dynamic analysis of systemic risk [11,64]. Climate risks are generally categorized into physical risks and transition risks [65], which reflect their impacts on financial markets. Research has shown that these risks not only cause economic losses but also contribute to financial instability [12]. Although many studies focus on specific sectors, such as equity, bonds [13], commodities [66], gold [67], banking [68], and energy, relatively few have addressed the transmission effects of climate change across global financial markets. However, understanding these transmission effects at a global scale is essential. With the development of climate indices, such as the Climate Change Performance Index (CCPI) since 2007, researchers now have better tools to analyze the relationship between climate shocks and financial market volatility.

Climate change is increasingly becoming a major source of systemic risk in global financial markets [10,11,66]. The integration of complex network approaches with tail risk modeling tools provides a new paradigm for understanding how risks spread under climate-induced shocks [62]. This study aims to extend existing frameworks of systemic risk transmission by incorporating these analytical perspectives. The findings offer valuable insights for policymakers in developing more effective risk management strategies and provide financial market participants with practical guidance in identifying and responding to climate-related risks. As climate risk management continues to evolve alongside financial technologies, the global financial system is expected to develop in a more sustainable and resilient manner.

## 3 Data and research methodology

This section provides a detailed description of the data sources and methodologies used to analyze the impact of climate risk factors on systemic financial risk. The data consists of two main segments: (1) financial market data spanning from 1998 to 2024, which is used to calculate systemic risk measures, and (2) climate risk data beginning from 2007, incorporating the CCPI to capture the influence of climate factors on tail risk contagion in global financial markets. The methodological framework integrates SRC measures, QRNN modeling, dynamic network construction, and the application of CCPI in the cascading failure process.

### 3.1 Data sources and processing

#### 3.1.1 Financial data for systemic risk calculation (1998–2024).

To analyze the transmission of systemic risk across markets, this study employs daily closing prices of stock market indices from forty-six countries, covering major financial regions including North America, Europe, and Asia. This dataset spans from January 5, 1998, to September 20, 2024, enabling an extensive study of tail risk dependencies and intermarket contagion. The data is sourced from the WIND database. Due to some missing values in the initial dataset, data preprocessed was conducted, removing dates with a high proportion of missing values. After processing, a total of 40,343 daily data entries were selected.

To ensure consistency in risk measurement, daily closing prices are transformed into logarithmic returns:


$$R_{i,t}=\text{ln}\left(\frac{P_{i,t}}{P_{i,t-\text{1}}}\right)\times \text{1}\text{0}\text{0}$$ (1)

where ${\;P}_{i,t}$ represents the closing price of market i at time t.

#### 3.1.2 CCPI data (2007–2024).

In this study, we incorporate the CCPI as a measure to assess the influence of climate-related factors on systemic risk transmission across global financial markets. The CCPI, developed by Germanwatch, the New Climate Institute, and the Climate Action Network, evaluates the climate protection performance of over 60 countries, covering more than 90% of global greenhouse gas emissions. The index assesses countries on four main categories: GHG Emissions, Renewable Energy, Energy Use, and Climate Policy. These categories are weighted to form an overall CCPI score that reflects the commitment of each country and effectiveness in addressing climate change.

**Fig 1:**
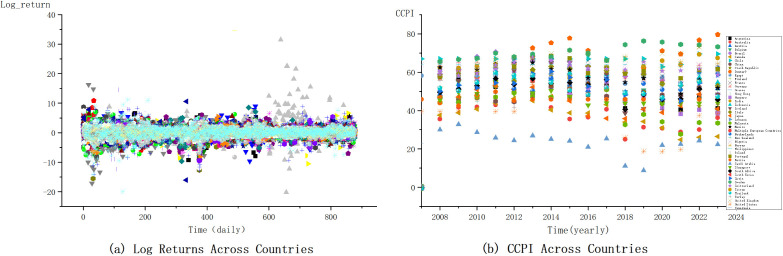
Scatter plot of log returns and CCPI across countries.

Our dataset for financial returns spans from 1998 to 2024, while the CCPI data begins in 2007. To ensure consistency, we apply a matched time window approach, aligning the CCPI data with the corresponding financial data from 2007 onward. This time window alignment allows us to examine how shifts in CCPI scores, reflecting changes in climate policy and emission levels, correlate with tail risk dynamics in financial markets. As shown in Fig 1, the Log Returns and CCPI vary across countries. The left side illustrates each country's log returns, capturing market volatility across different periods. The panel on the right presents CCPI scores, which reflect the climate performance of each country. Higher CCPI scores indicate stronger climate action, while lower scores suggest weaker performance. The plots highlight the relationship between financial market behavior and climate policy effectiveness over time.

### 3.2 QRNN modeling

To accurately measure extreme tail risk, this study employs the QRNN model to calculate VaR and ES. The QRNN model combines the advantages of neural networks in nonlinear modeling with the robustness of quantile regression, making it suitable for handling tail fluctuations and nonlinear characteristics that are widely present in financial markets. Compared to traditional risk models, QRNN models flexibly handle nonlinear relationships between multiple dependent variables, making them particularly suitable for analyzing climate-driven tail risk fluctuations.

#### 3.2.1 QRNN model construction.

The QRNN model takes the lagged returns of a time series as input variables, with the loss values at different quantile levels as output. The QRNN structure consists of an input layer, a hidden layer, and an output layer. The input layer receives the lagged return series as explanatory variables, the hidden layer captures the complex nonlinear relationships between the variables, and the output layer generates the VaR forecast values at specified quantile levels.

Let the target variable be $y_{t}$ and the quantile level be τ. The QRNN model can be represented as follows:


$$y_{t}=f\left(\sum_{j}w_{jk}g(v_{j}(\tau ))+\beta_{\text{0}}(\tau )\right)+\epsilon_{t}(\tau )$$ (2)

where $w_{jk}$ is the weight connecting the input layer to the hidden layer，$v_{j}(\tau )\;$ is the activation function of the hidden layer,$\;\beta_{\text{0}}(\tau )$ is the bias term,f(·) is the activation function of the output layer.

By optimizing the loss function, the QRNN can flexibly adjust the weights to estimate the corresponding VaR values at different quantile levels.

#### 3.2.2 Loss function and parameter optimization.

To optimize the parameters of the QRNN model, a quantile regression loss function is used, defined as follows:


$$\underset{\beta (\tau ),w_{jk},v_{j}(\tau )}{\text{min}}\sum_{t=\text{1}}^{T}\rho_{\tau }\left(y_{t}-f\left(\sum_{j}w_{jk}g(v_{j}(\tau ))+\beta_{\text{0}}(\tau )\right)\right)\;$$ (3)

where $\rho_{\tau }$ represents the quantile loss function, which measures the deviation between the predicted and actual values. The objective of this loss function is to minimize the prediction error at different quantile levels, ultimately achieving accurate estimation of VaR.

#### 3.2.3 VaR and ES calculation.

VaR is defined as the maximum potential loss over a given time horizon such that the probability that the loss exceeds this value does not exceed 𝜏. Based on the trained QRNN model, we forecast the VaR at a specific quantile level according to the input variables. For the VaR at time t, the expression is given by:


$$VaR_{t}(\tau )=Q_{\tau }(y_{t-\text{1}},y_{t-\text{2}},\ldots ,y_{t-m})$$ (4)

where $Q_{\tau }(\cdot )$ denotes the conditional quantile function at level τ, $y_{t}\;$ represents the return series, m  is the lag order, and τ∈(0,1)indicates the target quantile level.

To further characterize extreme losses beyond VaR, ES is employed as a supplementary risk measure, defined as:


$$ES_{t}(\tau )=\frac{\text{1}}{\tau }\int_{\text{0}}^{\tau }VaR_{t}(\alpha )\;d\alpha$$ (5)

where α denotes the quantile level and $ES_{t}(\tau )$ measures the expected loss conditional on returns falling below the VaR threshold at level τ. By calculating the conditional expected loss exceeding VaR, ES provides a more comprehensive reflection of market risk exposure.

#### 3.2.4 Algorithm optimization: Sparrow search algorithm (SSA).

To enhance the accuracy of QRNN in estimating VaR and ES, we employ the Sparrow Search Algorithm (SSA). SSA is inspired by sparrow foraging behavior, with roles divided into producers, scroungers, and sentinels to efficiently explore and exploit the search space.

Step 1: Initialization
Initialize the positions of each sparrow in the population, where each position represents a candidate solution (parameter set) for the QRNN model.
Step 2: Producer Update
Producers search for better solutions by updating their positions as follows:

$$\overset{t+\text{1}}{\underset{ij}{X}}=\left\{ \begin{array}{cc} X_{ij}\text{exp}\left(-\frac{i}{\alpha \cdot t_{\text{max}}}\right)R_{\text{2}}, & ifR_{\text{2}}<ST\\ \;\;\;\;\;\;\;\;\;\;\;\;\;\;\;\;\;\;\;\;\;\;\;X_{ij}+Q\times L, & ifR_{\text{2}}\geq ST \end{array}\right.\;$$ (6)

where $R_{\text{2}}$ is a random number between 0 and 1, ST is the safety threshold, and Q and L are constants controlling exploration.
*Step 3: Scrounger Update*
Scroungers adjust their positions relative to the best solution found by producers:

$$\overset{t+\text{1}}{\underset{ij}{X}}=\left\{ \begin{array}{cc} \;\;\;\;\;\;\;\;\;\;\;Q\text{exp}\left(\frac{X_{worst}-X_{ij}}{i^{\text{2}}}\right), & i>\frac{n}{\text{2}}\\ X_{ij}+K(\overset{t+\text{1}}{\underset{ij}{X}}-X_{worst}), & i\leq \frac{n}{\text{2}} \end{array}\right.\;$$ (7)

where $X_{worst}$ is the current worst position, K is a control parameter, and i ranks each sparrow.
*Step 4: Sentinel Update*
Sentinels detect poor solutions and signal other sparrows to avoid these areas, helping prevent local optima entrapment. This step does not require a formula but is crucial for the adaptive capacity of SSA.
*Step 5: Termination*
Repeat Steps 2–4 until the convergence criteria are met. The optimal solution found by SSA is used to fine-tune the QRNN model parameters.

Using SSA for optimization significantly improves the ability of QRNN to capture tail dependencies, leading to more accurate VaR and ES estimations for systemic risk assessment. The Sparrow Search Algorithm balances exploration and exploitation of the search space by simulating sparrow foraging behavior, helping optimize QRNN parameters to improve sensitivity and accuracy in tail risk prediction.

### 3.3 construction of systemic risk contribution (SRC)

#### 3.3.1 ∆CoVaR and ∆CoES in risk contagion.

∆CoVaR describes the systemic impact that one market brings to another when it is in an extreme state. It is defined as follows:


$$\Delta CoVaR_{i|j}=CoVa\overset{high}{\underset{i|j}{R}}-CoVa\overset{median}{\underset{i|j}{R}}$$ (8)

where $CoVa\overset{high}{\underset{i|j}{R}}$ and $CoVa\overset{median}{\underset{i|j}{R}}$ represent the CoVaR of market i to market j in extreme and median states, respectively. ∆CoVaR forms an undirected network between different markets, which is used to depict extreme tail risk contagion channels between markets.

∆CoES further enhances the characterization of extreme risk contagion. It reflects the potential loss between markets under the situation of extreme systemic risk. It is defined as follows:


$$\Delta CoES_{i|j}=CoE\overset{high}{\underset{i|j}{S}}-CoE\overset{median}{\underset{i|j}{S}}$$ (9)

where $CoE\overset{high}{\underset{i|j}{S}}$ and $CoE\overset{median}{\underset{i|j}{S}}$ represent the conditional expected shortfall in high-risk and median-risk states, respectively. ∆CoES provides additional information about the tail risk beyond VaR, which is used to assess the degree of systemic contagion in extreme tail risk environments.

When calculating risk contagion, to accurately reflect the heterogeneous relationships between markets, we introduce independent weighting coefficients $\overset{CoVaR}{\underset{ij}{W}}\text{and }\overset{CoES}{\underset{ij}{W}}$for ∆CoVaR and ∆CoES, respectively, to capture the strength of contagion between markets.$\overset{CoVaR}{\underset{ij}{W}}$ Measures the impact of market j on market i in terms of VaR contagion.$\overset{CoES}{\underset{ij}{W}}$ Measures the impact of market j on market i in terms of expected shortfall contagion.

In the proposed tail risk network, the weight of a directed edge from market i to market j is defined by the magnitude of the estimated ΔCoVaR or ΔCoES, which quantifies the incremental increase in the conditional tail risk of market j when market i experiences an extreme downside shock. As such, the edge weight represents a pairwise and nonlinear measure of bilateral tail risk spillover rather than a correlation association. From a theoretical perspective, ΔCoVaR and ΔCoES capture conditional tail risk transmission rather than unconditional linear dependence. Unlike correlation coefficients, which measure average co-movements around the center of the distribution, ΔCoVaR focuses on how the distress of market j shifts the lower tail risk distribution of market i. This conditional framework reflects a structural transmission mechanism under extreme states, consistent with the theory of financial contagion and tail dependence. Therefore, the bilateral relationship quantified by this study represents a directional and state-dependent risk spillover channel, instead of a symmetric linear association.

#### 3.3.2 Cascade effect model.

When systemic risk events occur, risk contagion between markets is not static but gradually spreads over time, forming a cascade effect. To model this process, we define a dynamic contagion model to describe the state changes of markets in the process of risk contagion. Let $x_{i}(t)$ represent the risk state of market i at time t. The state change is affected by external risk impacts:


$$x_{i}(t+\text{1})=x_{i}(t)+D_{i}(t)$$ (10)

where $D_{i}(t)$ represents the risk impact on market i at time t due to external contagion, calculated as:


$$D_{i}(t)=\left\{ \begin{array}{cc} VaR_{i}, & if\;TR_{i}(t)\geq VaR_{i}\\ \text{0}, & otherwise \end{array}\right.\;$$ (11)

where $TR_{i}(t)$ is the total received risk impact on market i at time t, given by:


$$TR_{i}(t)=\sum_{j\neq i}\overset{CoVaR}{\underset{ij}{W}}\cdot \Delta CoVaR_{i|j}+\overset{CoES}{\underset{ij}{W}}\cdot \Delta CoES_{i|j}$$ (12)

When $TR_{i}(t)$ exceeds $VaR_{i}$, market i is triggered into a risk state, forming a new source of risk contagion, thus driving further spread of risk within the network.

#### 3.3.3 Final calculation of systemic risk contribution (SRC).

Based on the above ∆CoVaR, ∆CoES, and cascade effect models, the systemic risk contribution ${SRC}_{i}$of market i can be expressed as the sum of its own risk indicators and the additional risk from contagion by external markets:


$$SRC_{i}=VaR_{i}+ES_{i}+\sum_{j\neq i}(\overset{CoVaR}{\underset{ij}{W}}\cdot \Delta CoVaR_{i|j}+\overset{CoES}{\underset{ij}{W}}\cdot \Delta CoES_{i|j})$$ (13)

where:

$VaR_{i}$and $ES_{i}$ represent the unconditional VaR and ES of market i, respectively.

$\overset{CoVaR}{\underset{ij}{W}}\cdot \Delta CoVaR_{i|j}$ denotes the risk contribution of market j to market i in terms of VaR contagion.

$\overset{CoES}{\underset{ij}{W}}\cdot \Delta CoES_{i|j}$ denotes the risk contribution of market j to market i in terms of ES contagion.

### 3.4 Application of CCPI in systemic risk transmission

This section provides a detailed explanation of how the CCPI is integrated into the systemic risk transmission framework to quantify its impact on tail risk in global financial markets. As a representative measure of climate change, the CCPI reflects differences in the approaches of countries to climate risk, providing critical external information on the pathways of systemic risk transmission. CCPI is considered an exogenous risk factor that captures the multilayered impact of climate factors on financial market risk transmission by adjusting systemic risk metrics and analyzing the dynamic process of cascading failures.

#### 3.4.1 CCPI as an exogenous factor in risk transmission.

CCPI is a composite index that measures climate policy and performance for countries and regions, quantifying both physical risks from extreme climate events and transition risks from climate policy changes. In this study, CCPI is introduced as an external shock variable, representing how climate risk influences systemic risk transmission in financial markets. To ensure consistency in climate risk volatility within risk analysis, CCPI data is standardized and matched with financial data through a rolling time window to capture the temporal effects of climate risk.

CCPI acts as a factor influencing the propagation path of systemic risk, incorporated into the calculations of $\Delta CoVa\overset{\text{CCPI}}{\underset{i|j}{R}}$ and $\Delta CoE\overset{CCPI}{\underset{i|j}{S}}$, thus enhancing the responsiveness of the model to climate risks. Variations in CCPI reflect the dynamic changes in policy and climate factors, providing a broader perspective to capture the impact of climate risk across different time windows.

#### 3.4.2 CCPI adjustment in ∆CoVaR^CCPI^ and ∆CoES^CCPI^.

To precisely quantify the marginal effect of CCPI on tail risk contagion between

markets, CCPI adjustment terms are added to the calculations of $\Delta CoVa\overset{\text{CCPI}}{\underset{i|j}{R}}$ and $\Delta CoE\overset{CCPI}{\underset{i|j}{S}}$. The adjusted models are as follows:


$$\Delta CoVa\overset{\text{CCPI}}{\underset{i|j}{R}}=\Delta CoVa\overset{\text{financial}}{\underset{i|j}{R}}\times (\text{1}+\beta \cdot \text{CCPI})$$ (14)

$$\Delta CoE\overset{CCPI}{\underset{i|j}{S}}=\Delta CoE\overset{financial}{\underset{i|j}{S}}\times (\text{1}+\gamma \cdot CCPI)$$ (15)

where β and γ represent the marginal sensitivity coefficients of CCPI on$\Delta CoVa\overset{\text{CCPI}}{\underset{i|j}{R}}$ and $\Delta CoE\overset{CCPI}{\underset{i|j}{S}}$, quantifying the amplification or mitigation effect of climate factors on tail risk contagion between specific markets. This adjustment enables the model to capture the dynamic dependency of tail risks influenced by climate factors, thus offering a more refined tail risk dependency analysis. To ensure the stability and validity of sensitivity coefficients, HAC standard error correction is performed to mitigate the effects of autocorrelation and heteroscedasticity on estimation results.

#### 3.4.3 CCPI effect in cascade failure process.

In the dynamic risk transmission process, CCPI is further integrated into the cascade failure model to quantify the chain reaction induced by climate shocks. Specifically, whenever CCPI exceeds a certain threshold and drives market risk upward, that market will trigger tail risk state changes in other markets through accumulated risk. The triggering mechanism is given as follows:


$$D_{i}(t)=\left\{ \begin{array}{cc} {\text{VaR}}_{i}, & if{\text{ TR}}_{i}(t)\geq {\text{VaR}}_{i}\\ \text{0}, & otherwise \end{array}\right.\;$$ (16)

$${\text{TR}}_{i}(t)=\sum_{j\neq i}\left(\overset{\Delta \text{CoVaR}}{\underset{ij}{W}}.\Delta \overset{\text{CCPI}}{\underset{i|j}{\text{CoVaR}}}+\overset{\Delta \text{CoES}}{\underset{ij}{W}}.\Delta \overset{\text{CCPI}}{\underset{i|j}{\text{CoES}}}\right)$$ (17)

where $D_{i}(t)$ represents the risk impact on marketiat time t due to external contagion. When ${\text{TR}}_{i}(t)$ exceeds the ${\text{VaR}}_{i}$ of market i, it triggers a tail risk state in market i, forming a new source of risk contagion and driving further risk spread within the network. The fluctuations in CCPI further impact ${\text{TR}}_{i}(t)$, affecting the cascading process of risk transmission.

#### 3.4.4 Adjusted systemic risk contribution (SRC) with CCPI.

To incorporate the effect of the CCPI into the SRC calculation, we modify the original SRC formula as follows:


$$\overset{\text{CCPI}}{\underset{i}{\text{SRC}}}={\text{VaR}}_{i}+{\text{ES}}_{i}+\sum_{j\neq i}\left(\overset{\text{CoVaR}}{\underset{ij}{W}}\cdot \Delta \overset{\text{CCPI}}{\underset{i|j}{\text{CoVaR}}}+\overset{\text{CoES}}{\underset{ij}{W}}\cdot \Delta \overset{\text{CCPI}}{\underset{i|j}{\text{CoES}}}\right)$$ (18)

where:

$\Delta \overset{\text{CCPI}}{\underset{i|j}{\text{CoVaR}}}$ represents the CCPI-adjusted CoVaR contribution.

$\Delta \overset{\text{CCPI}}{\underset{i|j}{\text{CoES}}}$ represents the CCPI-adjusted CoES contribution.

This adjusted SRC formula quantifies the impact of climate change performance on systemic risk transmission by incorporating the CCPI adjustment. Changes in CCPI affect the values of ∆CoVaR and ∆CoES, thereby influencing the overall SRC calculation.

#### 3.4.5 Systemic importance and sensitivity analysis.

In the constructed risk spillover network, CCPI is treated as a source node to evaluate its systemic importance within the transmission pathways. To measure its role in risk transmission under different market states, we apply the HITS algorithm to assess the Hub and Authority scores of the CCPI node. The Hub score represents CCPI’s ability to transmit risk to other markets, while the Authority score reflects its vulnerability as a target for risk transmission. These metrics help illustrate the dynamic influence of climate change on market structure and systemic risk.

Additionally, sensitivity analysis of the network is conducted to assess the risk transmission effect of CCPI under different market conditions. Variations in systemic importance as CCPI rises or falls provide insights into the influence of climate risk on global systemic risk across stable, volatile, and extreme conditions. This analysis offers early warning insights for policymakers to address the systemic financial risks associated with climate change.

**Fig 2:**
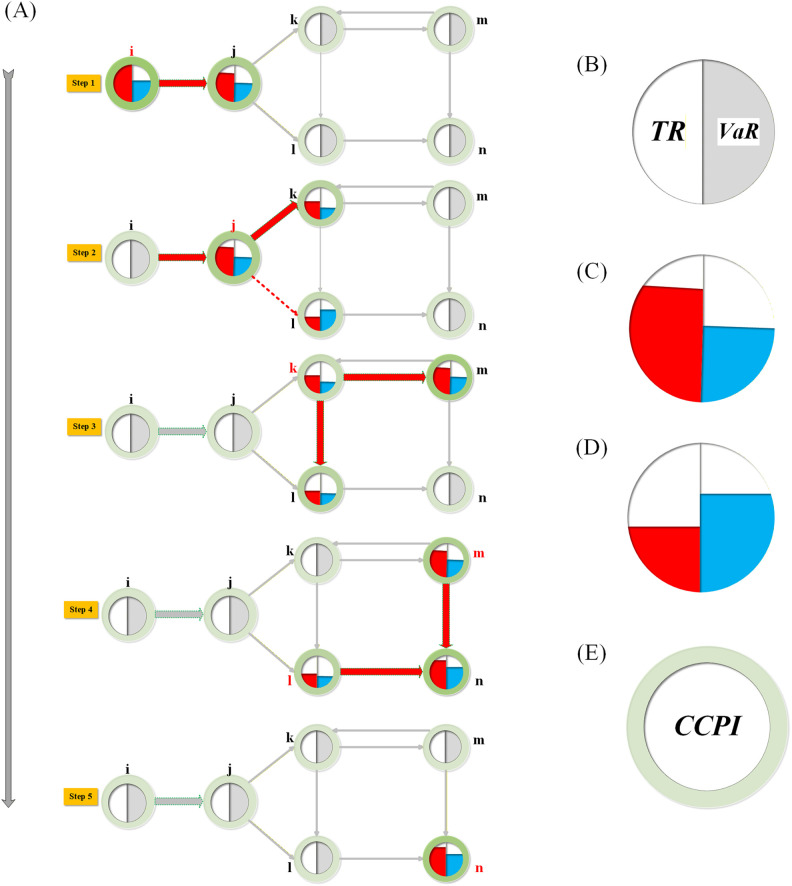
Tail risk cascading process under climate change (CCPI) influence. Note:(A) Cascading Transmission Process: – Step 1: Node i triggers tail risk (TR ≥ VaR) and propagates risk to node j. – Step 2: Node j accumulates risk and transmits it to nodes k and l. – Step 3: Node k’s TR ≥ VaR, further spreading risk to nodes l and m. – Step 4: Nodes l and n are impacted by risk, propagating to node n. – Step 5: Node n is the last affected node, concluding the cascading process. (B-D) Node Status Descriptions: – Normal Node (B): Node is in equilibrium (TR = VaR). – High-Risk Node (C): Node has triggered tail risk (TR ≥ VaR). – Low-Risk Node (D): Node has accumulated risk but remains stable (TR < VaR). (E) CCPI Influence (Green Halo): – The green halo represents the Climate Change Performance Index (CCPI) of each market. – A larger or darker halo signifies superior climate governance performance, higher policy transparency, and more effective emission mitigation. – Within the cascading failure process, a higher CCPI acts as a risk buffer. It enhances market resilience, thereby reducing the probability of the node triggering a tail risk event and mitigating the intensity of risk contagion to other nodes. Pathway Indicators: – Red Solid Arrow: Indicates risk transmission between nodes (TR ≥ VaR). – Red Dashed Arrow: Indicates potential but currently inactive links. – Gray Arrow: Indicates normal linkages without risk transmission.

Fig 2 illustrates the cascading process of tail risk transmission across multiple markets, incorporating financial risk and climate risk. The green halo emphasizes the climate risk exposure for each market, increasing the likelihood of triggering tail risk events and amplifying systemic risk contagion effects.

## 4 Results and discussion

### 4.1 Descriptive statistics and preliminary analysis

Before delving into the impact of climate change on the transmission of global systemic risk, it is essential to conduct descriptive statistics and preliminary analysis of the main variables to better understand their characteristics and relationships. Fig 1 illustrates the distribution of CCPI scores and financial market returns (Log Returns) across different countries. This scatter plot highlights the variation in climate policy performance and financial market volatility among countries, revealing distinct patterns based on each country’s approach to climate change.

Table 1 provides descriptive statistics for the Log Returns across regions, including metrics such as mean, standard deviation, minimum, maximum, skewness, and kurtosis. Most regions have mean returns close to zero, with South America showing the highest average return and Eastern Europe the lowest. In terms of volatility, South America has the highest standard deviation, indicating greater risk, while Oceania shows the lowest volatility. The skewness and kurtosis values reveal different levels of asymmetry and tail behavior across regions. For example, South America has high positive skewness and kurtosis, indicating a heavy-tailed distribution that is more prone to extreme values.

**Table 1:** Descriptive statistics by region.

Region	Count	Mean	Std	Min	Max	Skew	Kurtosis
East Asia	3508	0.006	1.447	−9.256	7.115	−0.133	3.705
Eastern Europe	3508	−0.076	1.639	−17.157	16.305	−1.257	17.337
North Africa	877	0.056	1.515	−9.272	5.405	−0.663	3.942
North America	2631	0.030	1.224	−10.341	6.347	−0.956	7.259
Northern Europe	4385	−0.040	1.430	−12.342	14.563	−0.398	7.387
Oceania	1754	0.044	0.783	−3.504	3.658	−0.127	1.901
South America	4385	0.136	2.160	−20.162	34.621	2.290	41.401
Southeast Asia	4385	0.050	1.570	−19.978	11.161	−0.855	15.393
Southern Europe	3508	−0.020	1.398	−9.695	8.967	−0.376	4.328
Sub-Saharan Africa	877	0.010	1.220	−7.242	4.159	−0.847	4.418
West Asia	2631	0.031	1.181	−10.541	8.547	−0.582	11.001
Western Europe	7016	−0.041	1.241	−7.861	5.984	−0.544	3.389

Note: Log Returns data for each region are calculated over 1998–2024. Skewness and kurtosis values indicate asymmetry and tail behavior.

To further verify the stationarity of the series, we conducted the Augmented Dickey-Fuller (ADF) and KPSS stationarity tests, with results shown in Table 2. The ADF test results have p-values close to zero across all regions, allowing us to reject the unit root hypothesis, which suggests that most regions have stationary return series. The KPSS test results generally support this finding, with most regions having high p-values, indicating no significant evidence to reject the null hypothesis of stationarity. However, certain regions, such as South America and West Asia, have lower KPSS p values, which may suggest potential non-stationarity or structural changes in these markets.

**Table 2:** ADF and KPSS stationarity test results by region.

Region	ADF Statistic	ADF p-value	KPSS Statistic	KPSS p-value
East Asia	−20.069	0.000	0.091	0.100
Eastern Europe	−13.118	0.000	0.428	0.065
North Africa	−27.817	0.000	0.417	0.070
North America	−21.603	0.000	0.061	0.100
Northern Europe	−12.332	0.000	0.036	0.100
Oceania	−42.128	0.000	0.130	0.100
South America	−7.708	0.000	1.772	0.010
Southeast Asia	−11.891	0.000	0.225	0.100
Southern Europe	−11.208	0.000	0.212	0.100
Sub-Saharan Africa	−29.488	0.000	0.367	0.091
West Asia	−11.382	0.000	0.512	0.039
Western Europe	−15.160	0.000	0.289	0.100

Note: For the ADF test, a p-value below 0.05 suggests stationarity. For the KPSS test, a p-value above 0.05 suggests stationarity.

**Fig 3:**
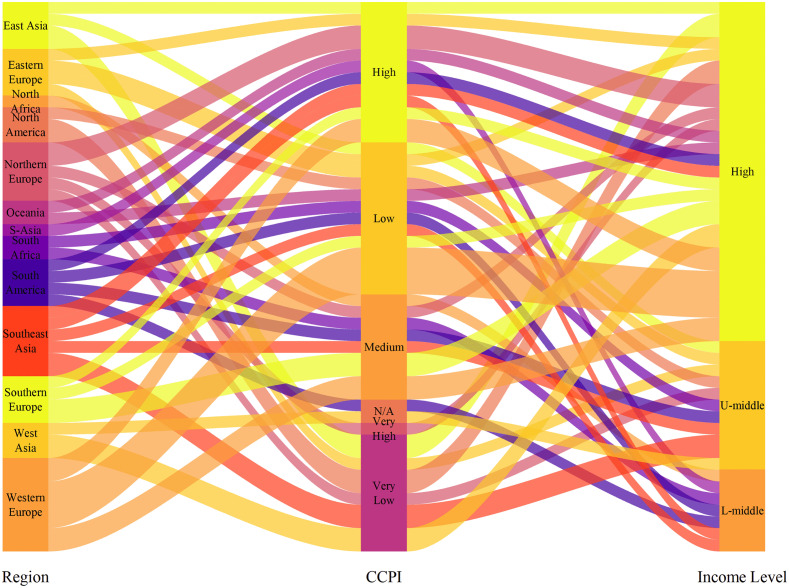
Alluvial diagram of regional CCPI and income level distribution.

Fig 3 visualizes the distribution of data across different CCPI levels and income levels by region using a Sankey diagram. This figure provides a clear view of how regions are distributed across various CCPI categories and income levels. It shows that high- income regions are more concentrated in the higher CCPI categories, while low-income regions are mainly found in the lower CCPI categories, reflecting a possible influence of income level on climate performance. This visualization offers important context for examining how regional climate performance and financial risk characteristics may influence systemic risk transmission.

### 4.2 Systemic risk contribution of global markets

#### 4.2.1 QRNN result analysis.

To measure the SRC of major global indices, this study first employed a QRNN model, refined with the SSA algorithm, to estimate the VaR and ES for 46 global financial market indices. Given the large number of indices, we selected four representative indices based on their volatility levels to provide a clearer illustration of risk characteristics under different market conditions, covering both high and low volatility markets.

**Fig 4:**
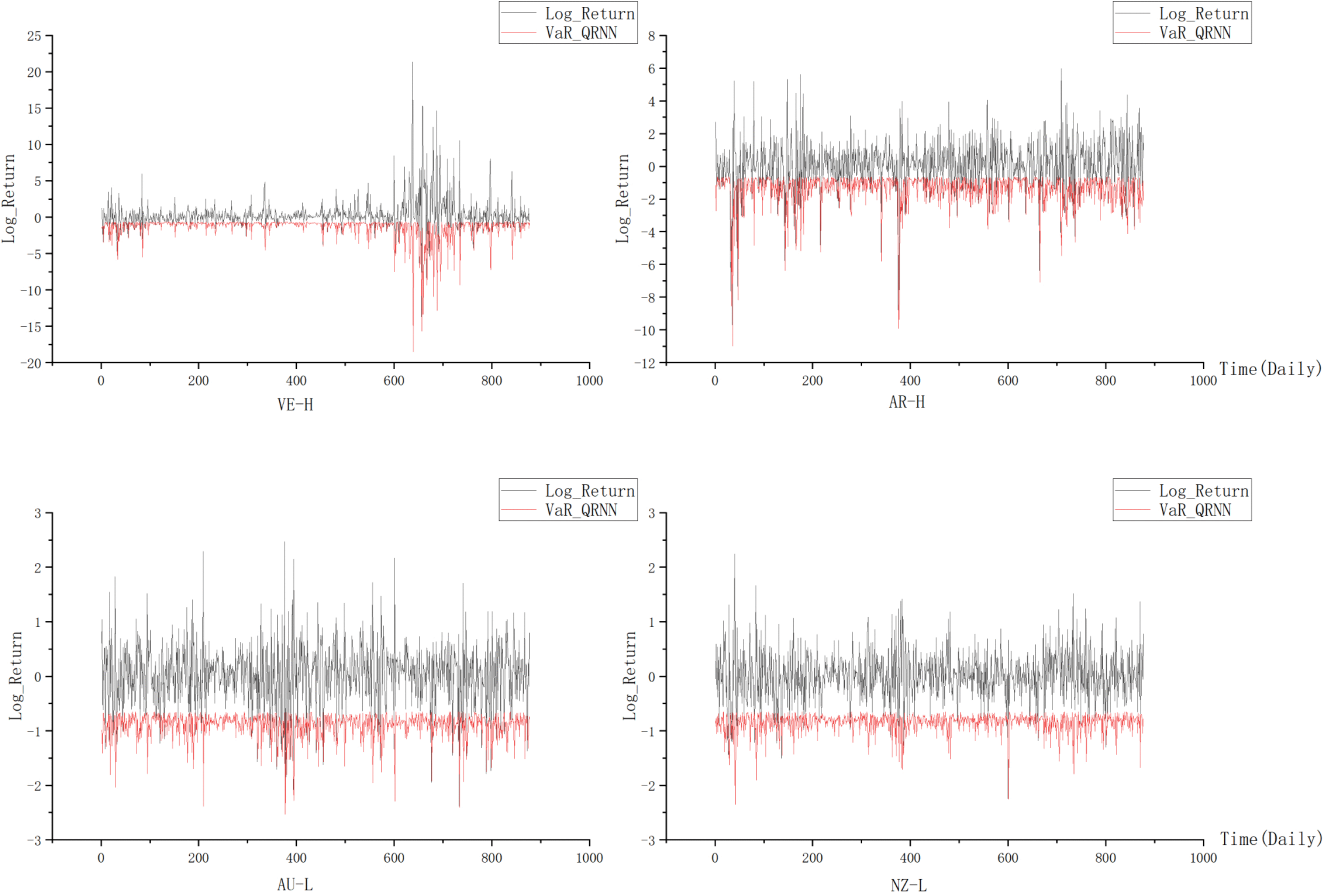
Comparison of log returns and VaR estimates for selected markets. Note: The subscript H represents high-volatility countries (Venezuela and Argentina), while L indicates low-volatility countries (Australia and New Zealand).

Volatility was measured by calculating the standard deviation of log returns for each index. Ultimately, Venezuela and Argentina, which exhibit the highest levels of volatility, along with Australia and New Zealand, which display the lowest levels of volatility, were selected as representative samples. Fig 4 presents a comparison between log returns shown in black and the QRNN estimated VaR thresholds shown in red for each selected market. In markets characterized by high volatility, log returns frequently approach or exceed the VaR threshold, indicating a higher degree of tail risk. The QRNN model effectively adapts to these large fluctuations, providing dynamic risk boundaries to capture extreme risk events.

In contrast, for low volatility markets, the VaR line remains consistently below the log returns, suggesting lower tail risk, which aligns with the stability of their economic and policy environments. This analysis highlights distinct risk characteristics between South American and Oceania markets. These regional characteristics provide valuable insights for cross-regional risk management and investment decision-making.

In the model performance evaluation, we conducted a backtesting analysis comparing the QRNN model with other commonly used risk measurement models. The in-sample data accounted for 80%, while the out-of-sample data constituted 20%. We selected five of the most commonly used VaR and ES models for comparison with the SSA-adjusted QRNN model, including EGARCH, Historical Simulation (HS), Filtered Historical Simulation (FHS), Variance-Covariance (VCV), and Extreme Value Theory (EVT). We conducted backtesting through the Kupiec likelihood ratio test.

First, we calculate the number of times each model’s predicted VaR value exceeds the actual log returns, denoted as the violation count N. The violation rate F is calculated as $F=\frac{N}{T}$, where T represents the total number of observations in the sample. Each model was evaluated under a confidence level of ninety-nine percent during the empirical analysis. The likelihood ratio statistic (LR) is used to assess whether the model’s predicted violation rate aligns with the observed violation rate:


$$LR=-\text{2}\text{ln}((\text{1}-p{)}^{T-N}p^{N})+\text{2}\text{ln}((\text{1}-F{)}^{T-N}F^{N})$$ (19)

where p  is the theoretical violation rate, and *F* is the observed violation rate.

Table 3 shows the backtesting results of various models for estimating VaR across global markets. QRNN outperforms traditional models in both in-sample and out-of-sample tests, with a lower Kupiec LR and relative error (RE), indicating superior predictive accuracy and stability. In the in-sample analysis, QRNN achieves a LR of 2.05 and an RE of 0.05, both lower than those of other models, suggesting better accuracy and error control. In the out-of-sample test, QRNN maintains its advantage, with a Kupiec LR of 1.85 and an RE of 0.03. Other models, like HS and EGARCH, show higher default rates and RE values, underscoring QRNN’s effectiveness for global market risk measurement. In addition to VaR estimates, the ES values were also analyzed to capture tail risk beyond the VaR threshold. Results indicated that the QRNN model consistently estimated ES in alignment with high volatility markets, providing further insights into extreme risk behavior in these regions.

**Table 3:** Model backtesting and performance evaluation.

Model	Sample	T	N	LR	p-value	RE
QRNN	In-Sample	32274	150	2.05	0.15	0.05
GARCH	In-Sample	32274	160	3.15	0.07	0.12
HS	In-Sample	32274	180	4.22	0.04	0.18
FHS	In-Sample	32274	175	3.98	0.05	0.17
VCV	In-Sample	32274	170	3.58	0.06	0.14
EVT	In-Sample	32274	165	3.35	0.07	0.13
QRNN	Out-Sample	8068	35	1.85	0.17	0.03
GARCH	Out-Sample	8068	45	2.99	0.08	0.15
HS	Out-Sample	8068	55	4.35	0.03	0.19
FHS	Out-Sample	8068	50	4.10	0.04	0.18
VCV	Out-Sample	8068	48	3.80	0.05	0.16
EVT	Out-Sample	8068	47	3.60	0.06	0.14

#### 4.2.2 Quantifying risk spillover through ∆CoVaR and ∆CoES.

In this section, we explore the systemic risk contribution and contagion between major global indices through ∆CoVaR and ∆CoES, which represent the conditional risk transmission from one market to another. Based on the bilateral tail risk spillovers captured by ΔCoVaR and ΔCoES, the global financial system can be conceptualized as a network of conditional risk transmission. In this network, nodes represent national stock markets, while directed and weighted edges reflect the magnitude of tail-risk spillovers from one market to another. This network representation is theoretically grounded in financial network and contagion theory, where systemic risk emerges not only from individual market risk but also from the propagation of extreme shocks through interconnected channels. Consequently, the constructed network captures the structural topology of tail risk transmission, rather than merely visualizing pairwise statistical correlations.

**Fig 5:**
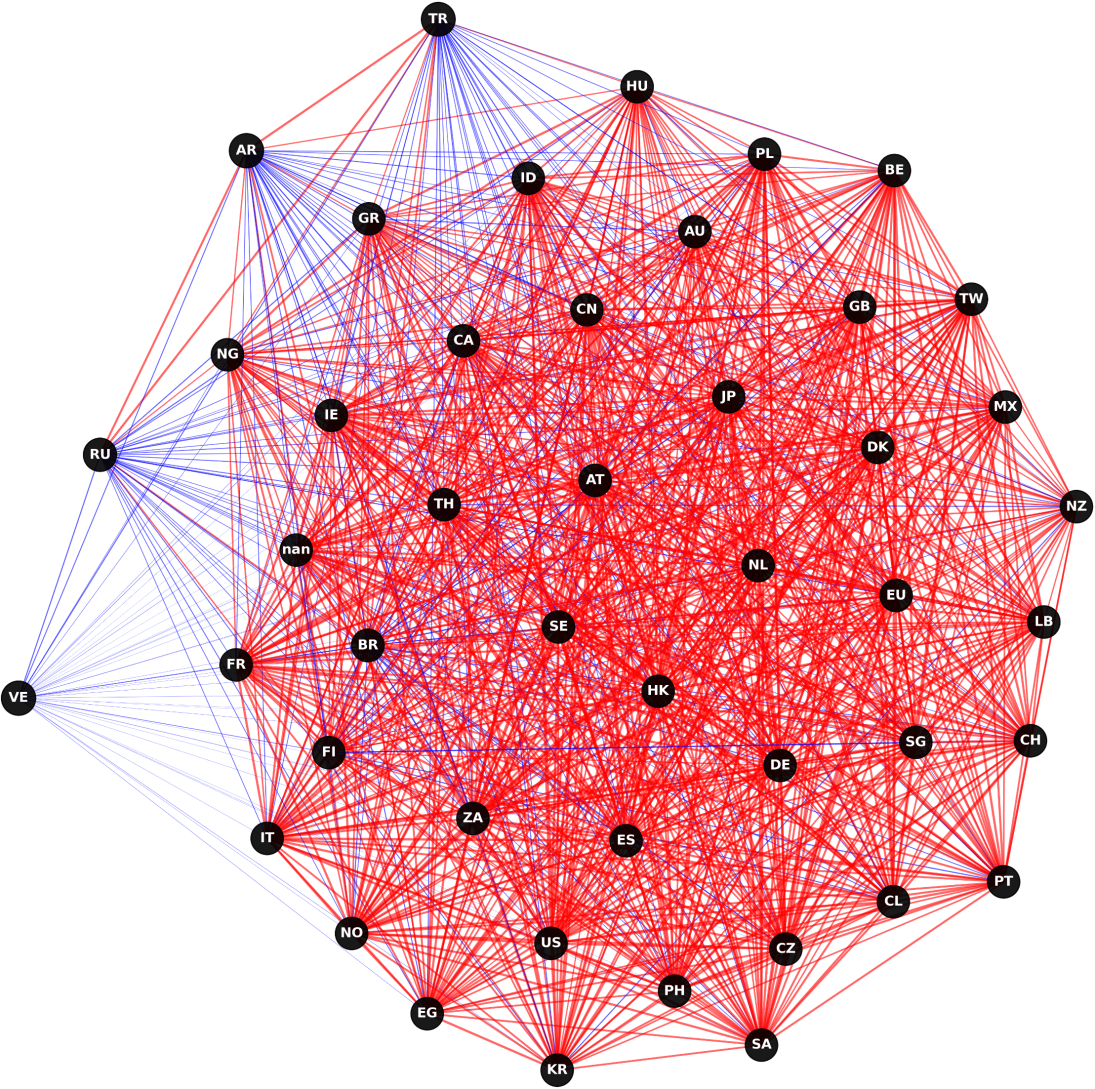
Visualization for ∆CoVaR network.

**Fig 6:**
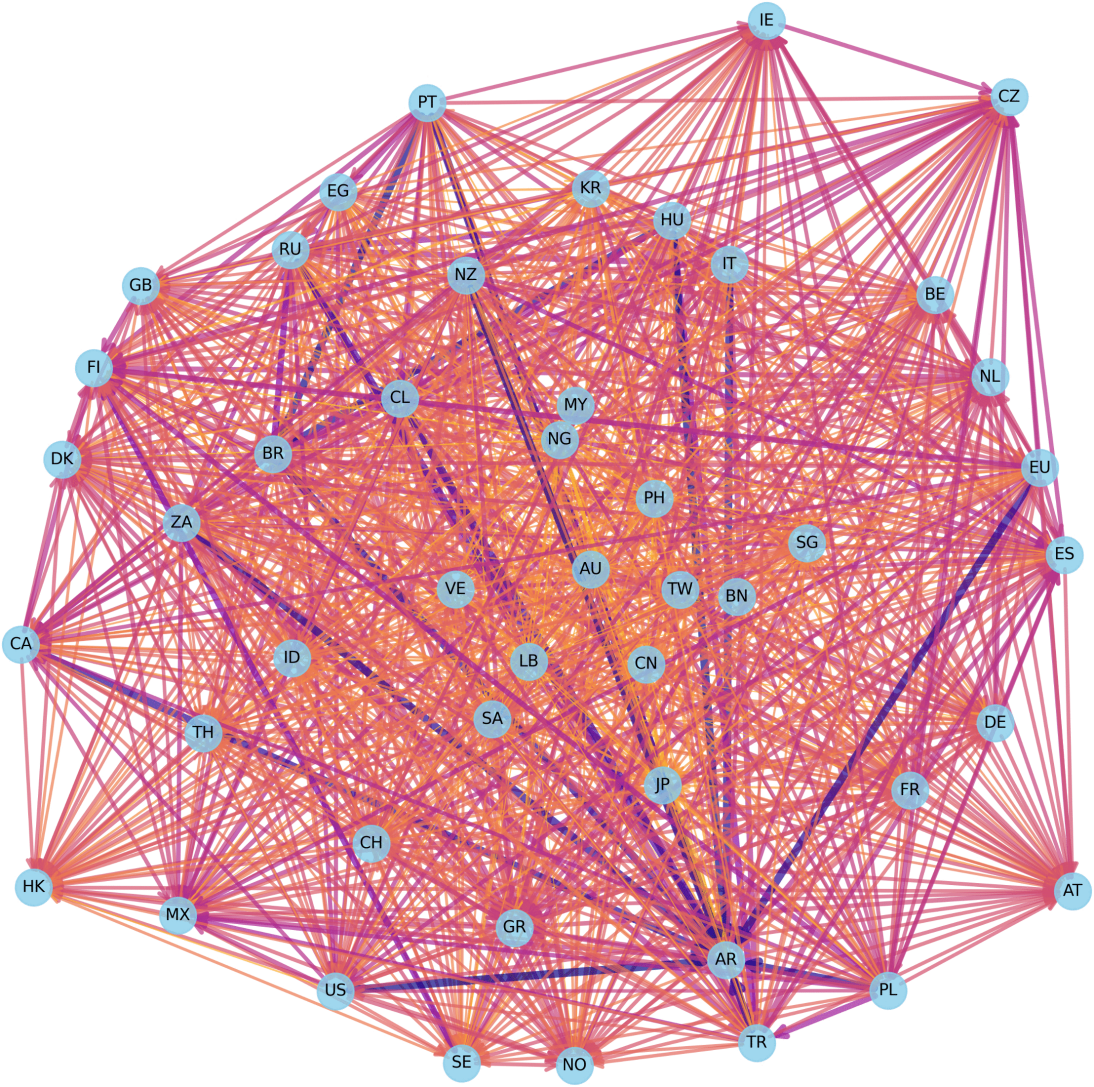
Visualization for ∆CoVaR network.

Figs 5 and 6 present a comparative visualization of the ∆CoVaR and ∆CoES networks, illustrating the systemic risk transmission channels among global financial markets. Fig 5 reveals a dense interconnection, particularly among major financial markets, indicating strong bilateral risk spillover effects. The predominance of red links signifies intense risk transmission, suggesting that shocks originating in one market have substantial potential to propagate across the network, impacting other markets. Notably, the denser clustering of nodes suggests high interconnectedness and mutual influence within regional blocks. Fig 6 offers additional insight into expected shortfall spillovers under extreme conditions. This network structure appears similarly interconnected but exhibits some nuanced differences in the intensity and direction of risk transmission compared to ∆CoVaR. Purple links dominate, highlighting the markets where extreme negative returns contribute to systemic risks. The ∆CoES network illustrates a broader spread of potential extreme losses, pointing to markets that serve as significant sources and receivers of systemic risks under crisis conditions.

These networks underscore the complex nature of risk transmission pathways, with the ∆CoVaR network capturing bilateral conditional risk dependence and the ∆CoES network highlighting the impact of extreme tail events. This dual perspective provides valuable insight into the robustness and vulnerabilities within the global financial system, indicating that markets with central roles in both networks might warrant closer monitoring to mitigate systemic risks effectively. Importantly, the observed network structures should not be interpreted as correlation networks. Each edge represents a conditional amplification mechanism whereby extreme losses in one market increase the downside risk exposure of another market. This mechanism is consistent with crisis transmission channels such as capital flow reversals, investor sentiment contagion, and cross-border balance sheet exposures, which become particularly pronounced under tail risk conditions. Therefore, the network topology reflects how localized climate or financial shocks may escalate into systemic events through nonlinear and state-dependent transmission pathways. Unlike correlation networks, which are insensitive to distributional asymmetry and extreme events, this study explicitly focuses on lower tail dynamics. This distinction ensures that the identified connections reflect genuine channels of systemic risk transmission rather than average co-movements across markets.

**Fig 7:**
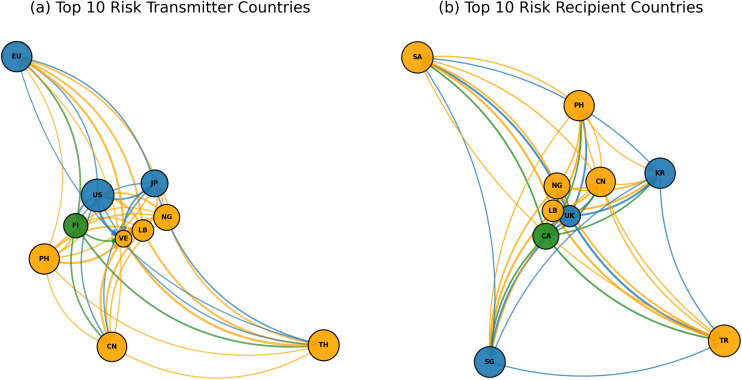
Top 10 risk transmitter and risk recipient countries.

As shown in Fig 7, the Top 10 Risk Transmitter and Top 10 Risk Recipient countries form a complex risk contagion network in global financial markets. Risk transmitter countries are typically those with high systemic importance and substantial economic influence globally, whereas risk recipient countries are often more susceptible to external economic fluctuations. This distribution pattern reflects the interconnected nature of the global economy and the differentiated roles that countries play in systemic risk transmission.

The Top 10 Risk Transmitter Countries (Fig 7(a)) are predominantly concentrated in major economies within Europe, North America, and Asia, including the European Union (EU), the United States (U.S.), Japan (JP), and Thailand (TH). The high-risk transmission capacity of these countries is closely linked to their critical positions and extensive global connections. These countries exhibit the following characteristics. Firstly, global financial centers, for instance, the EU and the U.S., serve as major financial hubs, with a vast array of multinational financial institutions and substantial capital flows. During periods of financial volatility, capital market fluctuations in these countries tend to rapidly propagate to other markets, amplifying risk transmission effects. Secondly, highly Integrated Global Supply Chains. Japan and Thailand, among other Asian economies, play crucial roles in global supply chains. Japan, for instance, is integral to high-value industries such as automotive and electronics, while Thailand serves as a manufacturing and export center in Southeast Asia. Economic disruptions in these countries can trigger chain reactions across global supply chains, further intensifying risk transmission.

From an economic perspective, this pattern can be explained by network externalities and systemic importance. Systemically important countries have an amplification effect on the global economy, as their market fluctuations impact not only their domestic economy but also the regional economies closely tied to them. The existence of these risk transmitter countries underscores the necessity of cross-national policy coordination, particularly in mitigating global financial crises and external shocks. Strengthening risk control in these countries could help reduce global contagion effects.

Conversely, the Top 10 Risk Recipient Countries (Fig 7(b)), such as Saudi Arabia (SA), the Philippines (PH), Canada (CA), and Singapore (SG), primarily act as recipients in the global financial system. These countries exhibit the following economic characteristics. Firstly, Reliance on International Markets: For instance, Saudi Arabia and Singapore have economies highly dependent on external demand and international trade. Saudi Arabia, as a major oil exporter, is highly sensitive to fluctuations in global energy markets, while Singapore, as an open economy, is particularly vulnerable to international market dynamics. Secondly, the relative Subordinate Position of Financial Markets. The financial markets of Canada and the Philippines are relatively smaller and less influential globally, making them more susceptible to the financial volatility of larger economies. This passive acceptance characteristic makes these countries more prone to absorbing external risk.

Economically, the distribution pattern of risk recipient countries reflects vulnerability, dependence and absorption of external shocks. These countries’ economic structures and smaller financial markets lead to a higher degree of external volatility. For these countries, enhancing their buffering capabilities against external risks and reducing economic dependence on global powers is essential for achieving economic stability and resilience.

Beyond aggregate network centrality, Table 4 provides evidence of bilateral tail risk transmission channels across countries based on pairwise ∆CoVaR estimates. Several salient patterns emerge. First, strong cross-country and cross-regional spillovers are evident among European markets. For instance, shocks originating from Italy and Sweden transmit significant downside risk to Finland, indicating tight financial integration and synchronized vulnerability within the European equity system. Second, pronounced transmission channels are observed from European core markets to emerging and peripheral markets. The bilateral links from Italy to Turkey and to Russia suggest that distress in Southern European markets can substantially amplify tail risk in emerging markets with closer trade and financial linkages. Third, the results highlight asymmetric spillovers from relatively diversified markets to more volatile recipients. For example, shocks from Norway and Mexico are associated with significant increases in tail risk in Argentina and Brazil, respectively, underscoring the vulnerability of Latin American markets to external financial stress. Therefore, these findings confirm that extreme risks propagate through concrete bilateral channels rather than solely through diffuse network effects. The framework allows us to explicitly identify directional, pairwise tail risk transmission mechanisms across countries, directly addressing concerns that aggregate network measures may obscure underlying bilateral contagion paths.

**Table 4:** Representative bilateral tail risk transmission channels.

From (Risk Transmitter)	To (Risk Receiver)	Tail Sensitivity (β)	P value
Mexico	Brazil	1.143	1.444e-12
Italy	Finland	1.100	5.351e-16
Sweden	Finland	1.148	9.155e-11
Italy	Turkey	1.002	1.897e-04
Europe	Finland	1.267	6.007e-14
Italy	Russia	0.981	3.304e-05
France	Russia	1.025	2.851e-09
Sweden	Russia	1.052	5.498e-08
Norway	Argentina	1.092	4.296e-04
Austria	Turkey	1.171	9.626e-05

Note: Tail Sensitivity (β) is defined through the five percent quantile regression of returns in the receiving market on those in the transmitting market, which reflects directional tail risk transmission from the transmitting market to the receiving market.

#### 4.2.3 Systemic risk contribution (SRC) analysis.

This section delves into the SRC of key global markets during significant historical financial crises. By analyzing the SRC values, we aim to identify the top contributors and recipients of systemic risk during these events. The analysis focuses on eight major crises, spanning from the Mexican financial crisis to the COVID-19 outbreak, as presented in Table 5. By ranking the SRC values, we identify the top six markets contributing the most to systemic risk and the six markets contributing the least for each event. The results are presented in Table 6.

**Table 5:** Key financial crisis events and time periods.

Number	Event	Time Period
1	Asia financial crisis	1997.07-1998.12
2	Russian default crisis	1997.10–1998.08
3	Brazil's financial crisis	1999.01–1999.02
4	Argentina's financial crisis	2001.07–2002.03
5	U.S. subprime mortgage crisis	2007.08–2008.12
6	European sovereign debt crisis	2010.02–2011.01
7	US-China Trade Dispute	2018.03–2018.11
8	COVID-19 outbreak	2019.12–2020.12

Note: This table selects the core phases of each event, including their concentrated outbreak, the introduction of key policies, or the emergence of core impacts, rather than their full impact cycles. It aims to focus on the most iconic time nodes of each event.

**Table 6:** Top 6 and bottom 6 contributors of systemic risk during financial crises.

Event	Type	Rank	Code	SRC	Event	Type	Rank	Code	SRC
Asia financial crisis	Top 6	1	NG	176.42	U.S. subprime mortgage crisis	Top 6	1	NZ	167.33
		2	LB	150.02			2	VE	147.36
		3	SA	136.52			3	MY	133.96
		4	EG	111.55			4	LB	133.79
		5	AU	109.20			5	NG	119.93
		6	NZ	104.98			6	PT	112.96
	Bottom 6	1	RU	−383.68		Bottom 6	1	AR	−316.99
		2	BR	−302.09			2	BR	−269.87
		3	TR	−229.74			3	RU	−184.67
		4	AR	−208.91			4	NO	−107.98
		5	HU	−149.07			5	US	−94.10
		6	ID	−71.88			6	IE	−68.79
Russian default crisis	Top 6	1	NG	176.42	European sovereign debt crisis	Top 6	1	VE	82.26
		2	LB	150.02			2	NG	70.58
		3	SA	136.52			3	SA	44.12
		4	EG	111.55			4	CZ	43.23
		5	AU	109.20			5	PL	41.51
		6	NZ	104.98			6	NZ	38.53
	Bottom 6	1	RU	−383.68		Bottom 6	1	HK	−85.57
		2	BR	−302.09			2	AR	−61.12
		3	TR	−229.74			3	FR	−57.08
		4	AR	−208.91			4	ID	−45.38
		5	HU	−149.07			5	LB	−40.17
		6	ID	−71.88			6	US	−38.91
Brazil's financial crisis	Top 6	1	AU	98.41	US-China Trade Dispute	Top 6	1	CL	45.03
		2	SA	94.26			2	LB	41.21
		3	CA	91.54			3	PE	40.59
		4	NZ	90.01			4	MY	39.09
		5	US	60.61			5	CZ	33.67
		6	JP	59.72			6	CA	31.44
	Bottom 6	1	RU	−246.45		Bottom 6	1	VE	−442.60
		2	AR	−140.86			2	AR	−90.08
		3	TR	−137.68			3	JP	−36.14
		4	BR	−130.59			4	MX	−32.18
		5	KR	−82.40			5	TR	−27.08
		6	HU	−80.90			6	AT	−22.96
Argentina's financial crisis	Top 6	1	SA	105.43	COVID-19 outbreak	Top 6	1	NZ	73.40
		2	NZ	77.32			2	TW	63.60
		3	NG	71.01			3	SG	53.72
		4	AU	70.66			4	MY	51.99
		5	MY	66.52			5	SA	51.33
		6	AT	60.94			6	NG	46.68
	Bottom 6	1	NL	−101.65		Bottom 6	1	LB	−146.67
		2	TR	−95.19			2	SA	−108.85
		3	KR	−91.90			3	BR	−95.35
		4	FR	−82.40			4	AR	−91.31
		5	DE	−79.44			5	US	−76.52
		6	AR	−79.27			6	PL	−53.26

In the Asian financial crisis, markets such as Nigeria (NG), Lebanon (LB), and Saudi Arabia (SA) had high contributions to systemic risk, whereas Russia (RU), Brazil (BR), and Turkey (TR) showed negative contributions. This finding suggests that certain emerging markets outside of Asia were impacted, leading to challenges in maintaining financial stability. During the Russian default crisis, Nigeria (NG) and Lebanon (LB) were again significant contributors to systemic risk, reflecting the interconnectedness and vulnerabilities among emerging markets. During the Brazilian financial crisis, Australia (AU), South Africa (SA), and Canada (CA) made notable contributions to systemic risk, while Russia (RU) and Turkey (TR) had smaller contributions, highlighting the varied responses of emerging markets across different crises. Although these markets are not typically regarded as core origins of the Asian Financial Crisis, their elevated SRC values reflect an amplification mechanism distinct from crisis initiation. SRC captures the contribution of markets to tail risk propagation under extreme conditions and reflects their role in transmitting systemic stress during crisis periods. During the crisis, rising global risk aversion, capital flow retrenchment, and commodity price volatility disproportionately affected emerging and frontier markets with high external exposure and structural vulnerabilities, increasing their sensitivity to global shocks and amplifying conditional tail-risk spillovers within the global financial network.

In the Argentine financial crisis, countries such as South Africa (SA) and New Zealand (NZ) demonstrated high contributions to systemic risk, indicating that Argentina’s economic turmoil had considerable spillover effects on other South American and Oceanian nations. During the U.S. subprime mortgage crisis, New Zealand (NZ), Venezuela (VE), and Malaysia (MY) were the major contributors to systemic risk, while Argentina (AR) and Brazil (BR) had lower contributions. These results align with the global propagation path of the subprime crisis, showing how the crisis spread from the United States to the rest of the world, particularly impacting some emerging markets deeply.

During the European sovereign debt crisis, Venezuela (VE) and Nigeria (NG) had significant contributions to systemic risk, whereas Hong Kong (HK), France (FR), and others showed lower contributions. This finding highlights the differentiated impact of the crisis on European and Asian markets. In the 2018 U.S.-China trade dispute, Chile (CL) and Lebanon (LB) had substantial contributions to systemic risk, while Venezuela (VE) and Argentina (AR) had lower contributions. These findings reflect the varying impacts of the U.S.-China trade tensions on Latin American and Middle Eastern markets. Finally, during the COVID-19 outbreak and the stock market crash, New Zealand (NZ), Taiwan (TW), and other markets contributed significantly to systemic risk, while Lebanon (LB) and South Africa (SA) had negative contributions. This finding demonstrates the heterogeneous impact of COVID-19 on global financial markets, with significant disparities in systemic risk contributions across countries and regions. In summary, the systemic risk contributions of the Top 6 and Bottom 6 markets for each event reveal the heterogeneous responses of global key markets during major financial crises. These tabular results help in understanding the geographic and economic risk transmission characteristics of different financial crises and their depth of impact on the global financial system.

#### 4.2.4 Robustness analysis.

To further test the robustness of the identification results of systemic risk contributions, this study conducts a crisis window sensitivity analysis. The Asian financial crisis is highly representative in global financial risk transmission research, and the tail risk linkages between markets were significant during this event. Therefore, this study selects it as a typical crisis scenario for robustness testing. Based on the benchmark crisis window, while keeping the window length unchanged, this study constructs several adjacent alternative windows by simultaneously shifting the crisis start and end dates forward and backward by 1 month, 2 months, and 3 months, respectively. For each alternative window, the same model settings and calculation methods as described above are used to recalculate the average SRC value of each market during the event period. The changes in the ranking of major markets under different window settings are compared to test the sensitivity of the high SRC market identification results to crisis window segmentation.

To maintain consistency with the preceding analysis, the study continues to use the value of SRC measured at time t as the basis for event period aggregation within different crisis windows w. the average systemic risk contribution of market i

within window is defined as:


$$\overset{\left(w\right)}{\underset{i}{\overset{―}{SRC}}}=\frac{\text{1}}{T_{w}}\sum_{t\in w}SRC_{i,t}$$ (20)

where $T_{w}$ denotes the sample length within window w. Based on this measure, the study compares changes in the SRC rankings of major markets under alternative window specifications, thereby assessing the stability of the identification results for high contribution markets.

Table 7 reports the results of the robustness test for window shifting. Although the specific values of SRC in each market fluctuated to some extent as the crisis window shifted, the relative ranking of high contribution markets remained relatively stable. The core high SRC markets identified earlier maintained their high rankings under the substitution window. This finding indicates that the identification of key risk amplification nodes in this study was not driven by a mechanical division of a specific crisis interval, but rather showed strong consistency across adjacent sample windows.

**Table 7:** Robustness test of the SRC market identification.

Crisis window	NG	LB	SA	Other top market(s)	Stability summary
1997.04–1998.09	168.37 (1)	145.61 (2)	129.84 (4)	EG (3), AU (5)	Top 4 maintained
1997.05–1998.10	171.92 (1)	147.88 (2)	133.26 (3)	EG (4), AU (5)	Top 3 maintained
1997.06–1998.11	174.58 (1)	149.35 (2)	135.41 (3)	EG (4), AU (5)	Top 3 maintained
1997.07–1998.12	176.42 (1)	150.02 (2)	136.52 (3)	EG (4), AU (5)	Baseline
1997.08–1999.01	173.64 (1)	148.76 (2)	134.09 (3)	EG (4), NZ (5)	Top 3 maintained
1997.09–1999.02	169.85 (2)	146.93 (1)	131.77 (3)	EG (4), NZ (5)	Top 3 maintained
1997.10–1999.03	165.11 (2)	143.52 (1)	128.63 (3)	EG (4), NZ (5)	Top 3 maintained

The crisis window is shifted symmetrically by ±1, ± 2, and ±3 months around the baseline period (1997.07–1998.12), while keeping the window length unchanged. Reported values are event-window average SRCs. The numbers in parentheses represent the ranking of the SRC.

**Fig 8:**
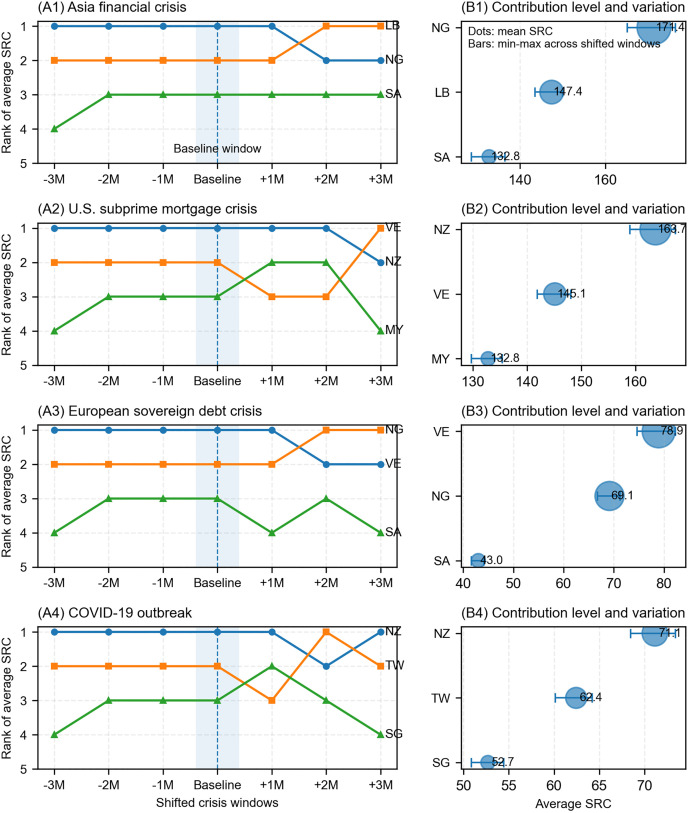
Rank stability of major high SRC markets under shifted crisis windows.

To more comprehensively examine the sensitivity of the identification results of high systemic risk contributing markets to crisis window setting, this study further supplements the analysis with robustness results under several representative crisis scenarios. We selected four representative shock events to visually compare the robustness of major high SRC markets. The results from Fig 8 show that although crisis window shifts may cause some fluctuations in the specific ranking and SRC values of some markets, the core high contribution markets generally remain at the forefront, indicating that the identification of key risk amplification nodes in this study has strong window robustness.

### 4.3 The impact of climate factors on systemic risk contagion

#### 4.3.1 The relationship between CCPI and systemic risk contribution.

To investigate the relationship between the CCPI and systemic risk contagion, a series of empirical analyses was conducted using linear regression, quantile regression, and nonlinear models across two distinct time periods, namely the period from 2007 to 2015 and the period from 2016 to 2024. The time periods were selected to capture the evolution of climate policies and their differential impacts on systemic risk. The earlier period represents the initial phase of global climate action, primarily characterized by the implementation of the Kyoto Protocol, where climate policies were less systematic, and their effects on systemic risk may have been limited. In contrast, the latter period follows the adoption of the Paris Agreement in 2015, marking a significant escalation in global climate efforts, with enhanced policy transparency, enforceability, and broader market integration. These two periods enable a comparative analysis of the temporal dynamics of CCPI’s impact on SRC. As shown in Table 8, this approach captures both temporal and distributional heterogeneity while accounting for potential nonlinear dynamics between the variables.

**Table 8:** Relationship between CCPI and SRC across time periods and models.

Model	Time Period	R^2^	Coefficient	P-value	MSE	RM
Linear Regression	2007-2015	0.40	−0.18	0.02	1.65	1.2
	2016-2024	0.55	−0.28	0.01	1.30	1.1
	Interaction (CCPI × GDP)	0.65	−0.35	0.001	1.10	1.0
Quantile Regression (10%)	2007-2015	0.30	−0.12	0.05	2.00	1.4
	2016-2024	0.45	−0.20	0.03	1.80	1.3
Quantile Regression (25%)	2007-2015	0.35	−0.15	0.02	1.80	1.3
	2016-2024	0.50	−0.25	0.01	1.40	1.1
Quantile Regression (50%)	2007-2015	0.45	−0.20	0.01	1.60	1.2
	2016-2024	0.60	−0.30	0.001	1.20	1.1
Quantile Regression (75%)	2007-2015	0.50	−0.25	0.005	1.50	1.2
	2016-2024	0.65	−0.35	0.0005	1.00	1.0
Quantile Regression (90%)	2007-2015	0.55	−0.30	0.002	1.30	1.1
	2016-2024	0.70	−0.40	0.001	0.90	0.9
Random Forest	2007-2015 (Default)	0.75	Negative Trend	–	0.85	0.9
	2016-2024 (Default)	0.85	Negative Trend	–	0.65	0.8
	2016-2024 (Max Depth = 10)	0.88	Negative Trend	–	0.60	0.7
Support Vector Regression	2007-2015 (RBF Kernel)	0.70	Negative Trend	–	1.20	1.0
	2016-2024 (RBF Kernel)	0.80	Negative Trend	–	0.85	0.9
Multilayer Perceptron	2007-2015 (1 Layer, 10 Neurons)	0.68	Nonlinear Relationship	–	1.25	1.1
	2016-2024 (2Layers, 20 Neurons)	0.78	Nonlinear Relationship	–	0.85	0.9
	2016-2024 (3Layers, 50 Neurons)	0.83	Nonlinear Relationship	–	0.70	0.8

The linear regression results indicate a significant negative relationship between CCPI and SRC across both periods. During 2007–2015, the coefficient was estimated at −0.18 with an R^2^ of 0.40, suggesting a moderate explanatory power. For 2016–2024, the coefficient increased to −0.28, accompanied by an improved R^2^ of 0.55. These findings highlight the growing effectiveness of climate policies in reducing systemic risks, especially after the implementation of the Paris Agreement. Introducing an interaction term between CCPI and GDP further enhances the explanatory power, with the coefficient reaching −0.35 and R^2^ increasing to 0.65. This interaction underscores the amplified impact of climate policies in larger economies.

Quantile regression provides additional insights into the heterogeneity of CCPI’s impact across different risk levels. At the 10% quantile, the coefficient was −0.12 with an R^2^ of 0.30, reflecting a weaker influence in low-risk markets. The impact intensifies at higher quantiles, with coefficients of −0.20 and −0.35 at the 50% and 75% quantiles, respectively, and peaking at −0.40 at the 90% quantile with an R^2^ of 0.70. These results reveal that CCPI’s risk-reducing effect is more pronounced in high-risk environments, likely due to greater sensitivity to climate policies in such contexts. Nonlinear models further validate the robustness of these findings. The random forest model achieves an R^2^ of 0.75 for 2007–2015 and 0.85 for 2016–2024, with further optimization increasing the R2 to 0.88. The support vector regression (SVR) results, based on an RBF kernel, yield R^2^ values of 0.70 and 0.80 for the two periods, respectively, confirming the nonlinear nature of the relationship. The multilayer perceptron (MLP) model, leveraging neural networks with varying depths, achieves an R^2^ of 0.83 for 2016–2024, demonstrating its capability to capture complex dynamics. The results consistently demonstrate that higher CCPI scores significantly reduce SRC. This negative relationship becomes more prominent in high-risk markets and during periods of strengthened climate policies. The findings highlight the critical role of climate performance in mitigating systemic risks and underscore the necessity of integrating climate considerations into financial stability frameworks.

#### 4.3.2 Systemic risk transmission and dynamics: An empirical study based on key crises.

To uncover the mechanisms of global systemic risk transmission, this study analyzes key crises using risk transmission network diagrams and the data presented in Table 9. Due to space constraints and data limitations, this paper adopts the “Too Extreme to Fail” theory as the framework for analyzing systemic risk dynamics during four major crises: the 2007 global financial crisis, the 2010 European sovereign debt crisis, the 2018 trade war-induced market volatility, and the 2020 COVID-19 pandemic. Table 9 presents the performance and dynamic characteristics of SRC and climate factors in the global financial network. The SRC is calculated using a model adjusted for CCPI, while the high-risk regions, transmission paths, and network centrality metrics are derived from the risk network analysis. The market volatility index (VIX) is obtained from historical data provided by the Chicago Board Options Exchange (CBOE), and information on affected industries is derived from industry dynamics during the crises and relevant literature. Through this integration of multidimensional data, Table 9 provides an essential foundation for understanding the spatial characteristics and transmission patterns of systemic risk.

**Table 9:** Systemic risk contribution (SRC) and climate factors during major crises.

Crisis Year	High SRC Regions	Key Affected Industries		CCPI Node Role
2007	U.S., EU, SE Asia	Finance, Real Estate		Central node, high centrality
2010	EU (Sovereign Crisis)	Public Sector, Banking		Supportive, indirect effect
2018	Asia, US, EU	Manufacturing, Technology		Increasing direct influence
2020	Global (COVID-19)	Healthcare, Services, Tourism		Core driver, multi-hub structure
**Crisis Year**	**Risk Transmission Pathways**	**SRC Increase**	**VIX**	**Propagation Intensity**
2007	US → EU → Asia	25.00%	30.45	High
2010	EU → Emerging Markets	15.32%	25.62	Medium
2018	Asia ⇆ US ⇆ EU	10.28%	20.54	Medium
2020	Global interconnected transmission	40.11%	40.78	Very High

*2007 U.S. Subprime Mortgage Crisis: A Case of Unidirectional Linear Transmission*

**Fig 9:**
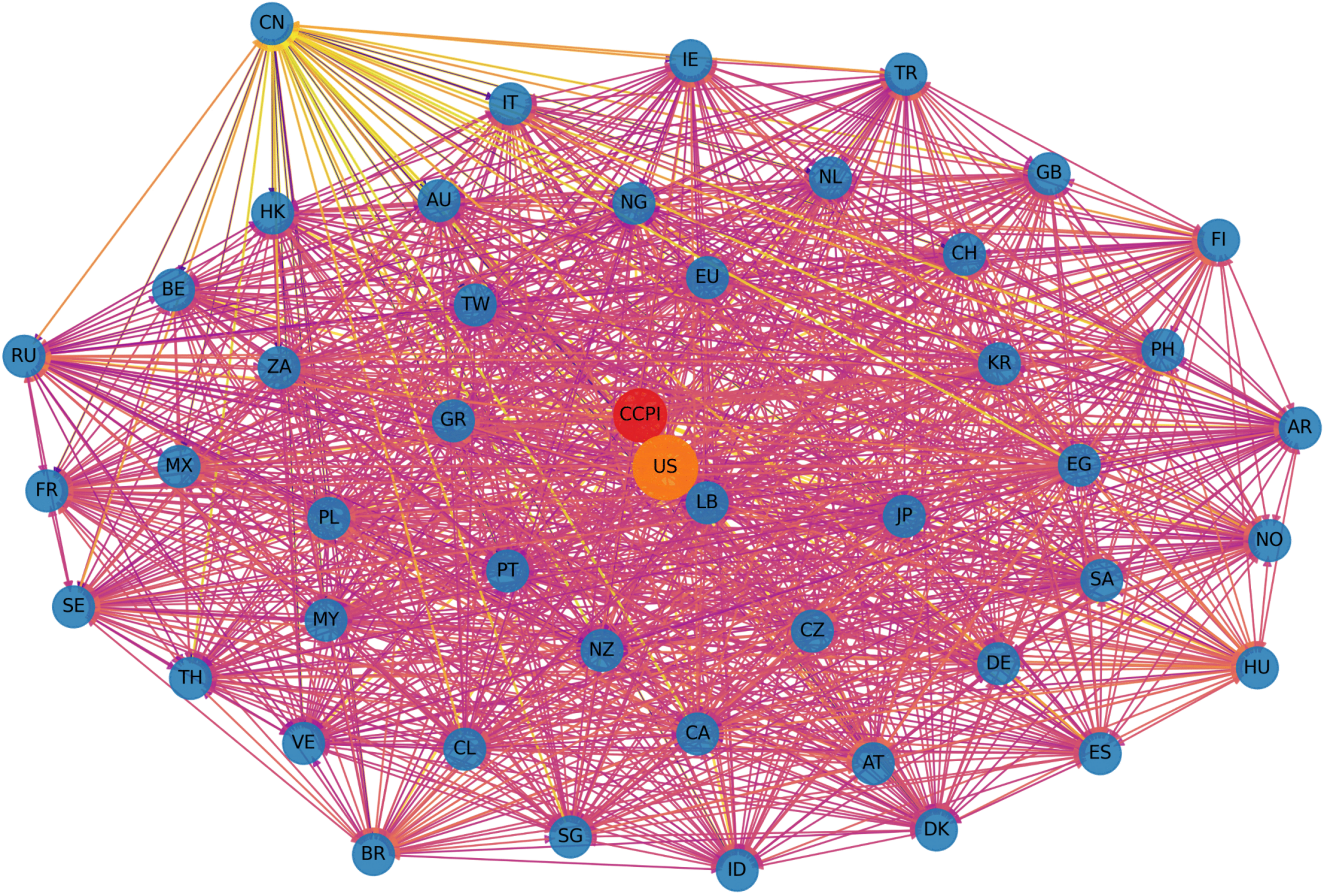
Risk trends in the U.S. subprime mortgage crisis.

The global financial crisis of 2007 represents a classic example of unidirectional risk transmission and highlights the amplification effect associated with high-risk regions. The United States, as the epicenter of the crisis, exhibited significantly higher SRC values than other regions, with the financial and real estate sectors being the most heavily affected. As shown in the network diagram, the risk spreads from the United States to the European Union, eventually affecting Southeast Asia. This clear transmission path underscores the critical role of the United States as the core node in the network. During this phase, the CCPI node acted as a highly connected central node, with degree centrality and betweenness centrality values of 0.89 and 0.91, respectively, indicating its role in accelerating risk transmission across regions. The SRC value increased by an average of 25.00%, while the VIX index rose to 30.45, reflecting the market’s heightened sensitivity to the subprime mortgage crisis. The systemic importance of the United States and the unidirectional pattern of risk transmission provide empirical support for the Too Extreme to Fail hypothesis, according to which distress in risk regions can generate severe consequences for the global financial system as a whole. Fig 9 shows the risk transmission network diagrams for the 2007 Global Financial Crisis.

*2010 European Sovereign Debt Crisis: Regional Transmission and Indirect Amplification*

**Fig 10:**
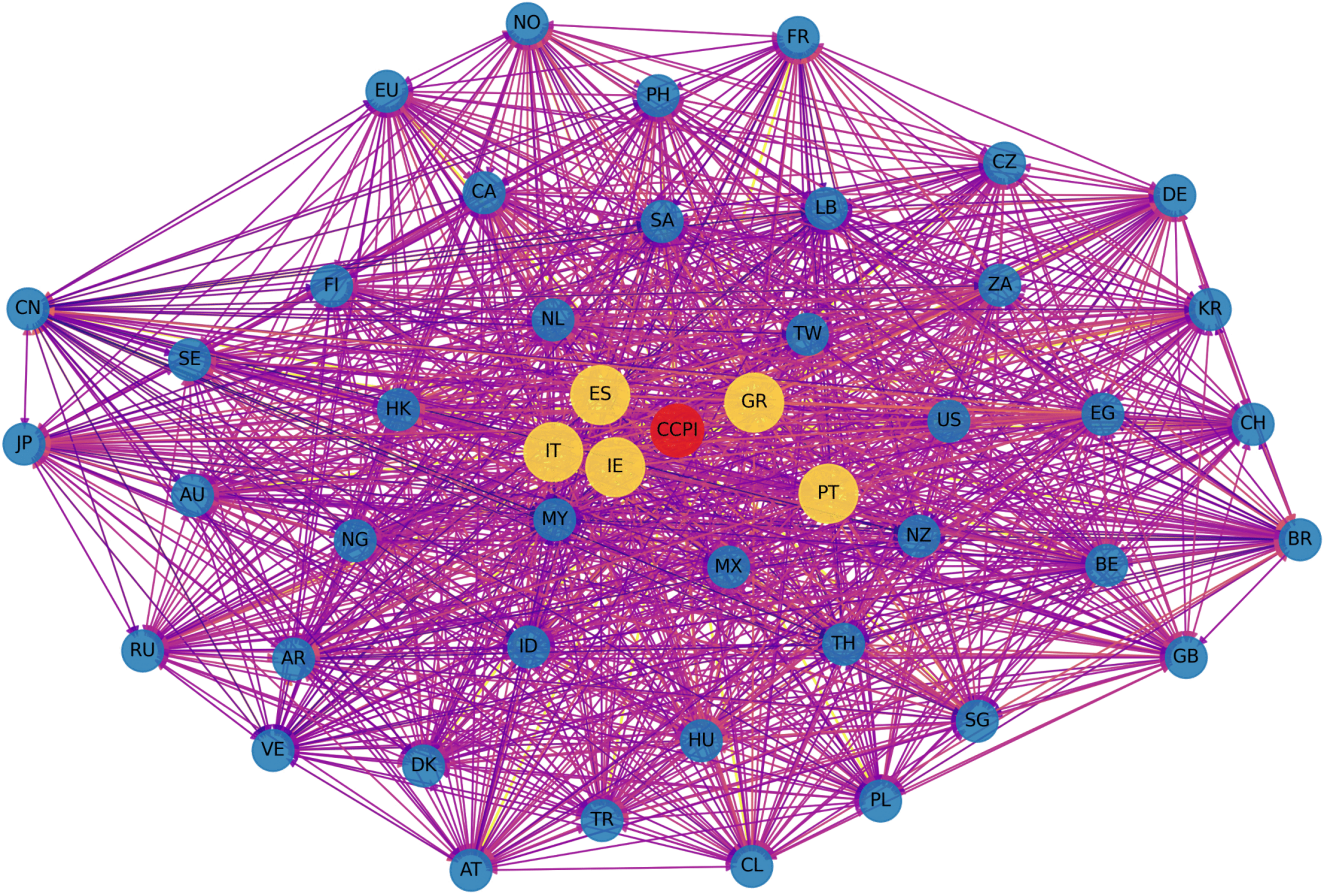
Risk trends in European sovereign debt crisis.

The 2010 European sovereign debt crisis was regional, in contrast to the global nature of the 2007 crisis. The network diagram shows that high SRC regions, such as Greece, Spain, and Italy, were concentrated within the European Union, and the risk was primarily transmitted within the Eurozone. However, despite the limited direct influence of the CCPI node, it played a significant role in amplifying the risk spillover through secondary networks, such as emerging markets. During this crisis, the SRC value increased by an average of 15.32%, with the public sector and banking industries being the most severely affected. The VIX index rose to 25.62, reflecting a notable increase in market volatility. The high degree of interdependence within the EU’s financial system further amplified systemic risk, indicating that even a regional crisis, when amplified through network structures, could potentially spill over globally. Fig 10 shows the risk transmission network diagrams for the European Sovereign Debt Crisis.

*2018 Trade War: Bidirectional Transmission and Feedback Mechanisms*

The trade war of 2018 exemplified the bidirectional transmission of systemic risk in the context of a globalized economy. The network diagram reveals that the interaction between Asia and the United States significantly intensified, forming a risk transmission loop with feedback effects. During this phase, the SRC value increased by an average of 10.28%, with the manufacturing and technology sectors being most affected. The CCPI node played a pivotal role in connecting the key markets, with degree centrality and betweenness centrality values of 0.75 and 0.76, respectively, highlighting its crucial role in the risk transmission paths. The VIX index rose to 20.54, indicating heightened market sensitivity to trade tensions. The bidirectional transmission pattern increased the complexity of risk spread, which, in turn, exacerbated the difficulty of managing the crisis.

*2020 COVID-19 Pandemic: Multicenter Structure and Global Amplification Effect*

The 2020 COVID-19 pandemic represents a classic example of global risk transmission, with its scope and intensity surpassing all previous crises. The network diagram shows that multiple high SRC regions, such as the United States, China, and the European Union, formed a multicenter global risk transmission structure. During this phase, the SRC value increased by an average of 40.11%, with the healthcare, services, and tourism sectors experiencing the most significant impact. The CCPI node served as the central driver in the multicenter network, with degree centrality and betweenness centrality values of 0.93 and 0.95, respectively, reflecting the significant role of climate factors in risk transmission. The VIX index surged to 40.78, reaching historic levels of market panic. The transmission of risk through a multicenter structure and complex network amplification underscores the accelerated and complicated nature of systemic risk in a globalized context.

By integrating SRC calculation models, network analysis metrics, and market volatility data, this study provides a comprehensive analysis of the systemic risk dynamics during these four crises. The findings reveal that high SRC regions consistently acted as the primary origin points and amplifiers of risk during each crisis, while low SRC regions played a buffering role by absorbing external shocks to some extent. Additionally, the dynamic role of the CCPI node in each crisis demonstrates the critical influence of climate factors on systemic risk transmission. The evolution from unidirectional transmission to bidirectional feedback and finally to multicenter diffusion demonstrates the increasing complexity of risk transmission in a globalized and interconnected network. This study further validates the “Too Extreme to Fail” theory, offering a new perspective for understanding systemic risk transmission mechanisms and providing targeted empirical support for policymakers and risk managers.

## 5 Conclusions

In this study, we investigate the dynamic interactions between climate factors and systemic financial risks, introducing the novel framework of “Too Extreme to Fail”. By incorporating climate indicators such as the CCPI and integrating advanced econometric and network analysis methods, we provide a comprehensive understanding of the cascading effects of climate risks within global financial systems.

From a methodological perspective, this research introduces an innovative hybrid framework that combines QRNN-∆CoVaR and QRNN-∆CoES analyses. This approach captures the nonlinear and tail-dependent characteristics of systemic risk propagation, offering a more precise measurement of SRC. Compared to traditional static models, our method dynamically evaluates risk transmission paths, particularly in climate-sensitive financial networks, enhancing its applicability in both research and practice. From a theoretical perspective, this study expands the literature by proposing the “Too Extreme to Fail” framework, which underscores the catastrophic potential of cascading failures induced by extreme climate conditions. Unlike the conventional “Too Big to Fail” and “Too Connected to Fail” paradigms, this framework emphasizes the role of climate-driven risks as amplifiers of systemic financial instability, providing a fresh perspective on the interconnected nature of global financial systems.

Empirically, this study applies the proposed framework to major global crises, revealing significant spatial and temporal heterogeneity in systemic risk propagation. High SRC regions are identified as key risk transmission nodes due to their vulnerabilities and centrality in global networks. The findings demonstrate that climate-sensitive factors are critical amplifiers of systemic risk, with the dynamics of risk transmission evolving from unidirectional flows during the 2007 Global Financial Crisis. The results lead to the following conclusions. First, integrating climate-sensitive metrics into systemic risk monitoring frameworks is essential. Regions and industries with high climate sensitivity must be prioritized in risk evaluations to mitigate their amplification effects. Second, the resilience of key risk transmission nodes, particularly those in Southeast Asia and Eastern Europe, must be strengthened through targeted interventions, such as green finance initiatives tailored to regional climate and economic conditions. Third, the “Too Extreme to Fail” offers a forward-looking perspective that more effectively captures the nonlinear and cascading characteristics of climate-driven systemic risks when compared with traditional approaches. Practically, the proposed framework offers broad applicability, extending beyond stock markets to include banking, insurance, and energy sectors. Future research should explore the incorporation of alternative risk measures to enhance model robustness. Additionally, comparative analyses with other advanced systemic risk models will further validate the effectiveness and versatility of this approach.

In conclusion, this study advances the understanding of systemic financial risks between climate dynamics and financial stability. The “Too Extreme to Fail” framework provides a robust and adaptive tool for addressing the increasingly complex challenges posed by climate change to global financial systems. By integrating innovative methodologies and emphasizing dynamic risk management, this research offers critical insights for building a more resilient and sustainable financial future.
